# Ribonucleotides embedded in template DNA impair mitochondrial RNA polymerase progression

**DOI:** 10.1093/nar/gkab1251

**Published:** 2022-01-08

**Authors:** Meenakshi Singh, Viktor Posse, Bradley Peter, Maria Falkenberg, Claes M Gustafsson

**Affiliations:** Department of Medical Biochemistry and Cell Biology, Institute of Biomedicine, University of Gothenburg, Gothenburg, SE-405 30, Sweden; Department of Medical Biochemistry and Cell Biology, Institute of Biomedicine, University of Gothenburg, Gothenburg, SE-405 30, Sweden; Department of Medical Biochemistry and Cell Biology, Institute of Biomedicine, University of Gothenburg, Gothenburg, SE-405 30, Sweden; Department of Medical Biochemistry and Cell Biology, Institute of Biomedicine, University of Gothenburg, Gothenburg, SE-405 30, Sweden; Department of Medical Biochemistry and Cell Biology, Institute of Biomedicine, University of Gothenburg, Gothenburg, SE-405 30, Sweden

## Abstract

Human mitochondria lack ribonucleotide excision repair pathways, causing misincorporated ribonucleotides (rNMPs) to remain embedded in the mitochondrial genome. Previous studies have demonstrated that human mitochondrial DNA polymerase γ can bypass a single rNMP, but that longer stretches of rNMPs present an obstacle to mitochondrial DNA replication. Whether embedded rNMPs also affect mitochondrial transcription has not been addressed. Here we demonstrate that mitochondrial RNA polymerase elongation activity is affected by a single, embedded rNMP in the template strand. The effect is aggravated at stretches with two or more consecutive rNMPs in a row and cannot be overcome by addition of the mitochondrial transcription elongation factor TEFM. Our findings lead us to suggest that impaired transcription may be of functional relevance in genetic disorders associated with imbalanced nucleotide pools and higher levels of embedded rNMPs.

## Graphical Abstract

**Graphical Abstract:**
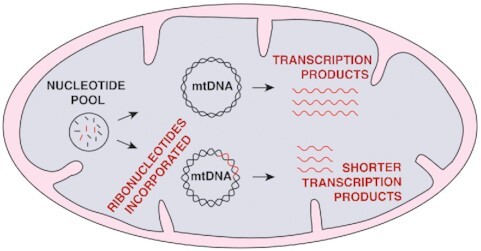
Ribonucleotides embedded in template DNA impair mitochondrial RNA polymerase progression.

## INTRODUCTION

The human mitochondrial genome (mtDNA) is a double stranded, 16.6 kb circular molecule that has >1000 copies in most somatic cells and encodes for 13 components of the oxidative phosphorylation system (1,2). Polycistronic transcription is initiated from two promoters, the light-strand (LSP) and heavy-strand (HSP), by the single subunit mitochondrial RNA polymerase (POLRMT) (1). To initiate transcription, POLRMT requires two accessory transcription factors, TFAM and TFB2M (3–7). The process begins with TFAM binding to a high-affinity site located 15–35 bp upstream of the transcription start site. POLRMT is then recruited via direct interactions with TFAM. Finally, TFB2M enters the complex, inducing structural changes in POLRMT that lead to promoter opening and trapping of the non-template DNA strand (8–13). Once initiated, transcription is further stimulated by the mitochondrial transcription elongation factor (TEFM), which forms a stable ternary complex with the elongating POLRMT and template DNA (14–17). TEFM helps POLRMT bypass structural elements in nascent RNA, such as the G-quadruplex forming region at conserved sequence box II (CSBII) located immediately downstream of LSP. TEFM also helps POLRMT to bypass 8-Oxo-dG lesions on the template strand (15,18).

Notably, mtDNA has high levels of embedded ribonucleotides (rNMPs), with 10–50 evenly distributed between the two strands of the mtDNA molecule (19,20,21). The ribonucleotides are misincorporated by mtDNA polymerase γ (POLγ) during DNA synthesis (21–23). The major nuclear replicative DNA polymerases (Pol α, Pol δ, and Pol ϵ) in eukaryotic cells also incorporate ribonucleotides during DNA synthesis, but to prevent genomic instability there are repair pathways that remove these embedded rNMPs (24). Ribonucleotide excision repair (RER) depends on RNase H2, which introduces a nick in double stranded DNA (dsDNA) at the 5′-side of the embedded rNMPs. A stretch of the incised strand is then replaced together with the rNMP, performed by Pol δ and the flap-endonuclease FEN1, and is followed by ligation. A second system for RER involving topoisomerase 1 (TOP1) can cleave DNA at embedded rNMPs (25–29).

Mitochondria lack known systems for RER (30). The pattern of RNA incorporation and the levels of embedded rNMPs are affected by the mitochondrial deoxyribonucleotide (dNTP) pools. When dNTP levels are limiting, POLγ is more likely to use ribonucleotides NTPs as building blocks (21). As a consequence, disease-causing mutations that alter nucleotide pools lead to changes in the levels of incorporated rNMPs (21). This is similar in other eukaryotes: analysis of yeast strains with altered dNTP pools revealed an inverse relationship between the concentration of individual dNTPs and the levels of the corresponding rNMPs incorporated in mtDNA (30).

Increased levels of embedded ribonucleotides in patient cells with disturbed nucleotide pools may induce a pathogenic mechanism that disturbs new rounds of mtDNA replication. Increased levels of embedded rNMPs in mtDNA are, for instance, observed in cell lines derived from patients with pathogenic variants of genes encoding enzymes involved in nucleotide metabolism, such as thymidine kinase 2, deoxyguanosine kinase, or MPV17 (21), and in yeast cells with disturbed nucleotide pools (30). POLγ can bypass single rNMPs with high fidelity and efficiency, but stretches of embedded rNMPs in the template strand cause replication stalling or termination. This may lead to mtDNA depletion and, eventually, to mitochondrial disease phenotypes (22,23).

POLRMT also uses mtDNA as a template, but it has not been determined whether embedded ribonucleotides disturb mitochondrial transcription. In this study, we use a series of *in vitro* experiments to address this point. We demonstrate that mitochondrial RNA polymerase activity is impaired by embedded rNMPs and discuss the possible mechanisms underlying this effect.

## MATERIALS AND METHODS

### Reagents

Unless otherwise stated, oligonucleotides were from Eurofins Operon. KOD polymerase and *Escherichia coli* DE3 pLysS Rosetta cells were from Novogen. RNase inhibitor murine and T7 RNA polymerase were from New England Biolabs. Glycogen was from Roche, Proteinase K from Invitrogen, and Heparin Sepharose from Sigma.

The rapid DNA ligation kit was from Thermo Scientific. QIAquick PCR purification, QIAquick Gel elution, and QIAquick nucleotide removal kits were from Qiagen. The QuikChange site-directed mutagenesis kit was from Agilent. A 2 ml HiTrap heparin column and Superdex™ 200 gel filtration column were from GE Healthcare Life Sciences.

### Template preparation

The ssDNA template preparation was adapted from a reported study (31). The wild-type RNA-primed DNA template was formed by annealing an RNA oligonucleotide (5′-UUU UGC CGC GCC; non-template RNA strand) to a DNA oligonucleotide (5′-GGG AAT GCA TGG CGC GGC; template strand). RNA-primed DNA templates were also formed with a series of different oligonucleotides containing embedded rNMPs at defined positions in the template strand. The embedded rNMPs are indicated in bold and underlined characters: 5′-GGG AA**U** GCA TGG CGC GGC (rNMP at position 17, R17U); 5′-GGG A**AU** GCA TGG CGC GGC (rNMPs at positions 17 and 18, R17–18UA); 5′-GGG **AAU** GCA TGG CGC GGC (rNMPs at positions 17–19, R17–19UAA); ribonucleotides at every third position 5′-**G**GG **A**AT **G**CA **U**GG CG CGGC (rNMPs at positions 13, 16, 19 and 22, Alt Ribo) (Eurofins Operon). RNA-primed templates were labelled with γ-^32^P ATP at the 5′-end. Free nucleotides were removed with QIAquick nucleotide removal kit as per manufacturer's instruction (Qiagen). Template strand (100 nM) was annealed with non-template strand (400 nM) at 95 °C for 5 min followed by slow cooling in a heating block.

To create double-stranded DNA constructs with embedded rNMPs in the template strand, we used a plasmid containing a fragment corresponding to position 471–638 of human mtDNA cloned in the *pUC*18 vector between BamHI and HindIII. With this plasmid as a template, we used PCR (KOD polymerase, Novogen) to amplify a 168 bp fragment containing the HSP promoter region. The amplicon formed included a 90 bp sequence upstream of the transcription start site (containing the TFAM binding site) and 78 bp downstream. The primers used for PCR amplification were designed with or without incorporated ribonucleotide(s) in the reverse-primer. The primer pair used to amplify the WT control fragment was as follows: 5′-TAC TAC TAA TCT CAT CAA TAC AAC CCC CGC C and 5′-GGT GAT GTG AGC CCG TCT AAA CAT TTT CAG TG (Eurofins Operon). Ribonucleotides were introduced in the reverse-primer as indicated in bold and underlined characters: 5′-GGT GAT GTG AGC CC**G** TCT AAA CAT TTT CAG TG (r1G); 5′-GGT GAT GTG AGC CC**GU**CT AAA CAT TTT CAG TG (r2GU); 5′-GGT GAT GTG AGC CC**GUC**T AAA CAT TTT CAG TG (r3GUC); 5′-GGT GAT GTG AGC CC**AAA**T AAA CAT TTT CAG TG (r3A); 5′-GGT GAT GTG AGC CC**GGG**T AAA CAT TTT CAG TG (r3G); 5′-GGT GAT GTG AGC CC**CCC**T AAA CAT TTT CAG TG (r3C); 5′-GGT GAT GTG AGC CC**UUU**T AAA CAT TTT CAG TG (r3U); and 5′-GGT G**A**T GT**G** AGC **C**CG T**C**T AA**A** CAT **U**TT C**A**G TG (rAlt). The PCR products were purified with the QIAquick PCR purification kit.

To prepare a dsDNA template containing ribonucleotides in the non-template strand downstream of HSP, we used a 160 nt template strand DNA oligo: 5′-ATC TCA TCA ATA CAA CCC CCG CCC ATC CTA CCC AGC ACA CAC ACA CCG CTG CTA ACC CCA TAC CCC GAA CCA ACC AAA CCC CAA AGA CAC CCC CCA CAG TTT ATG TAG CTT ACC TCC TCA AAG CAA TAC ACT GAA AAT GTT TAG ACG GGC TCA CAT CAC C-3′ (Eurofins Operon). The template strand was hybridised to two oligonucleotides (each 80 nt long) that were ligated together to form the complete non-template strand. One of the non-template strand oligonucleotides did not contain embedded rNMPs and was used for all constructs: 5′-GGT GAT GTG AGC CCG TCT AAA CAT TTT CAG TGT ATT GCT TTG AGG AGG TAA GCT ACA TAA ACT GTG GGG GGT GTC TTT GG-3′. The sequence of the second oligonucleotide varied, depending on the presence of embedded rNMPs: 5′- GGT TTG GTT GGT TCG GGG TAT GGG GTT AGC AGC GGT GTG TGT GTG CTG (WT); 5′-GGT GAT GTG AGC CCG TCT AAA CAT TTT CAG TGT ATT GCT T**UGA**GG AGG TAA GCT ACA TAA ACT GTG GGG GGT GTC TTT GG-3′ (3 rNMP incorporated oligo); 5′-GGT GAT GTG AGC CCG TCT AAA CAT TTT CAG T**G**T A**U**T G**C**T TTG AGG AGG TAA GCT ACA TAA ACT GTG GGG GGT GTC TTT GG-3′ (alternate rNMP incorporated oligo).

The template strand (100 nM) was annealed to the non-template strand oligonucleotides (400 nM) at 95 °C for 5 min followed by slow cooling in a heating block. The annealed oligonucleotides were purified by the QIAquick nucleotide removal kit, as per manufacturer's instructions (Qiagen). The annealed template was ligated using the Thermo rapid ligation kit at room temperature for 30 min. After ligation the templates were separated on a 2% agarose gel followed by elution using the QIAquick Gel elution kit as per manufacturer's instructions (Qiagen).

### *In vitro* transcription

Transcription with dsDNA templates was performed in 25 μl reactions containing 10 mM Tris–HCl (pH 8.0), 10 mM MgCl_2_, 10 mM DTT, 60 mM NaCl, 100 μg/ml bovine serum albumin (BSA), 400 μM ATP, 150 μM GTP, 150 μM CTP, 10 μM UTP, 0.02 μM α-^32^P UTP (3000 Ci/mmol), 4 U RNase inhibitor murine (New England Biolabs), 50 nM TFAM, 60 nM TFB2M, 20 nM POLRMT and, when indicated, 40 nM TEFM. To the reactions, 8 nM of template was added when ribonucleotides were present in template strand, and 16 nM of template when ribonucleotides were present in non-template strand. Reactions were first incubated without CTP and TEFM at room temperature for 2 min to allow the formation of the transcription initiation complex. After addition of CTP and TEFM (when indicated), the reactions were incubated at 32 °C for another 28 min to produce run-off (RO) transcription products. Reactions were stopped by addition of 5 μl concentrated stop buffer (4 mg/ml Glycogen (Roche), 1.2 M NaCl, 60 mM Tris–HCl (pH 8.0), 6 mM EDTA, 0.8 mg/ml Proteinase K (Invitrogen)).

Transcription reactions with T7 RNA polymerase (T7 RNAP) were performed as previously described (15), but with the following modifications. A 25 μl mixture of 0.02 μM α-^32^P UTP (3000 Ci/mmol), 8 nM indicated template, and 4 U T7 RNAP (New England Biolabs) was prepared. The reaction was incubated at 37 °C for 20 min before being stopped as described above.

Pulse-chase transcription experiments were performed as described above. An excess of cold 100 mM UTP (0.5 μl of per 25 μl of reaction), was added at the indicated time points.

For the RNA-primed template, the template was labelled at the 5′ end with γ-^32^P ATP. The reaction mixture contained 10 mM Tris–HCl pH (8.0), 10 mM MgCl_2_, 10 mM DTT, 60 mM NaCl, 100 μg/ml BSA, 100 μM ATP, 100 μM GTP, 100 μM CTP, 100 μM UTP, 4 U RNase inhibitor murine (New England Biolabs), 4 nM template, 80 nM POLRMT and, when indicated, 160 nM TEFM. The reactions were stopped after 30 s or 1 min by the addition of stop buffer as described before (15).

### Polyacrylamide gel electrophoresis and phosphor imaging

Experiments using RNA primed DNA templates were analysed on 12% denaturing polyacrylamide gels (1 × TBE-buffer and 7 M urea). For the dsDNA template, samples were analysed on 10% denaturing polyacrylamide gels (1 × TBE-buffer and 7 M urea). All of the gels were exposed to photo-film or visualized by Phosphor imaging.

The Phosphor imaging data were analysed with ImageJ version 1.53 (Supplementary Figures S1–S4) or Multigauge (Figures 2B, D, F, 4B, 5C, Supplementary Figures S5 and S6). GraphPad Prism 8 was used to generate the graphs.

### Site-directed mutagenesis

Mutant derivatives of human POLRMT were created by changing the amino acid at position 774 from serine to alanine (S774A) and at position 807 from tyrosine to phenylalanine (Y807F) using a QuikChange site-directed mutagenesis kit according to the manufacturer's instructions (Agilent). As a template for the reactions, we used the expression vector pTEV-MBP4 containing the POLRMT open reading frame inserted between Nde I and Hind III restriction sites.

### Protein expression and purification

Wild type and mutant derivatives of POLRMT were expressed in *Escherichia coli* DE3 pLysS Rosetta cells (Novogen). The pTEV-MBP4 vector containing the POLRMT open reading frame was expressed in Luria broth (LB). Expression was induced with 1 mM isopropyl b-D-thiogalactopyranoside (IPTG) at 16 °C for 18 h. Cells were harvested and lysed in 20 mM HEPES–KOH (pH 8.0), 250 mM NaCl, 1 mM EDTA and 0.1 mg/ml lysozyme. Initial purification was performed with heparin sepharose batch purification (Sigma) equilibrated with buffer A (0.2 M NaCl, HEPES–KOH (pH 8.0), 10% glycerol, 1 mM DTT and 1 mM EDTA) and eluted with buffer A containing 0.8 M NaCl. The maltose binding protein tag (MBP) was cleaved with homemade Tobacco Etch Virus (TEV) protease and removed with a 2 ml HiTrap heparin column (GE Healthcare Life Sciences) using buffer A. POLRMT eluted at ∼800 mM NaCl. Further purification was carried out using a gel filtration column (Superdex™ 200 GE Healthcare Life Sciences) equilibrated with the following buffer: 25 mM HEPES–KOH (pH 8.0), 0.5 mM EDTA, 0.4 M NaCl and 1 mM DTT.

TFB2M, TFAM and TFEM were expressed and purified as previously described (32).

## RESULTS

### Embedded ribonucleotides in the template strand stall transcription

**Figure 1.**
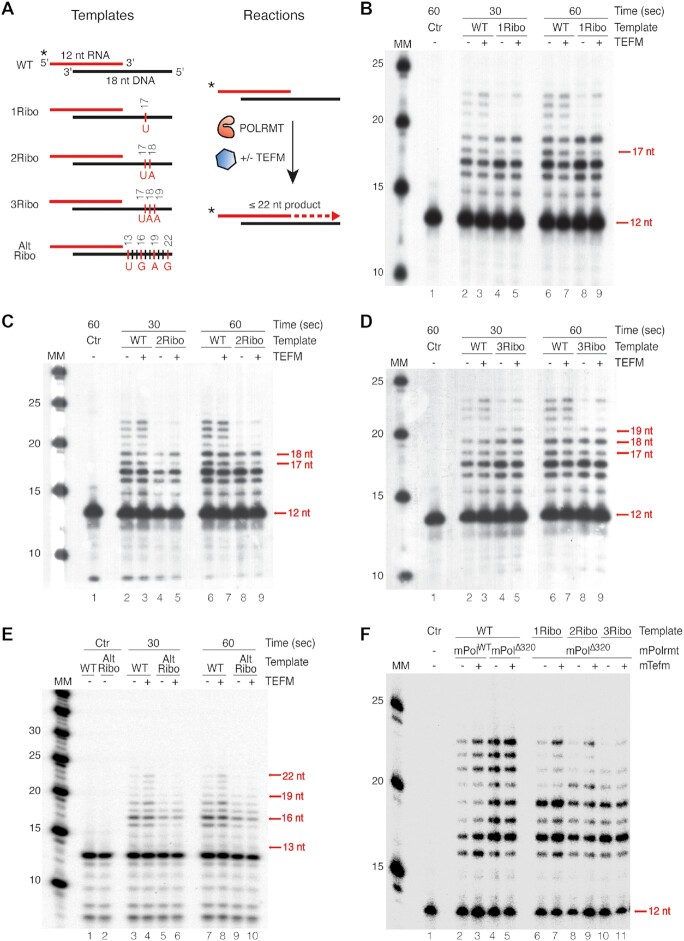
Transcription is stalled at ribonucleotides in the DNA template. (**A**) Schematic representation of the template; the 12 nt, 5′-labeled (^32^P) RNA strand annealed to the 18 nt DNA template strand. Run-off transcription creates a full-length product (22 nt). (**B**) *In vitro* transcription with **U**-ribonucleotide at position 17 (R17U). Lane 1 is a control with a WT template. POLRMT was added to all lanes except lane 1. TEFM was added as indicated. The reaction was carried out for 30 s in lanes 2–5 and for 1 min in lanes 1, 6–9. (**C**) *In vitro* transcription with **UA**-ribonucleotide at positions 17–18 (R17-18UA). Lane 1 is a control with a WT template. The reactions were performed as in (B). (**D**) *In vitro* transcription with **UAA**-ribonucleotide at positions 17–19 (Ribo17-19UAA). Lane 1 is a control with a WT template. The reactions were performed as described in (B). (**E**) *In vitro* transcription with an alternate ribonucleotide template. Lanes 1 and 2 are in the absence of POLRMT and TEFM, lanes 3–10 are as lane 2–9 are in (B). (**F**) *In vitro* transcription with mouse POLRMT and mouse TEFM performed with ribonucleotide embedded templates like (B), (C) and (D). Lane 2 and 3 were performed with mouse WT POLRMT and lane 4–11 were performed with mouse Δ 320 POLRMT.

To determine the consequences of rNMPs embedded in the DNA template strand for mitochondrial transcription, we used an RNA-primed DNA template containing a 12 nt RNA primer and an 18 nt DNA template. The two oligonucleotides formed an 8 bp duplex with a 4 nt 5′-RNA overhang and a 10 nt stretch of single-stranded DNA (Figure 1A). We incubated the template with POLRMT in the presence or absence of TEFM. Since the RNA template was primed, additional mitochondrial transcription factors (TFAM and TFB2M) required for promoter-specific transcription were not added (33).

When the template was incubated with POLRMT, the RNA primer was elongated and generated a 22 nt RO as well as multiple, shorter transcription products (Figure 1B, lane 2 and 6). The shorter transcription products are explained by the short incubation time and that POLRMT is non-processive on ssDNA templates (34). To test the ability of POLRMT to transcribe past embedded ribonucleotides, one or more rNMPs were incorporated into the template strand (Figure 1A). We first performed the experiment using a template with one rNMP at position 17 and in the presence of all four NTPs, and noted that POLRMT had problems progressing more than 1–2 nt past the embedded rNMP (Figure 1B, lanes 4 and 8). Addition of TEFM had no effect on the primed, single-stranded DNA template (Figure 1B, lanes 5 and 9). This failure of TEFM to stimulate POLRMT activity on a single-stranded template is in agreement with structural studies, which demonstrated that TEFM sequesters the non-template DNA strand and prevents the transcription bubble from collapsing (17).

Templates containing two or three rNMPs embedded in a row gave similar results (Figure 1C and D). We also performed a transcription reaction with a template with ribonucleotides at positions 13, 16, 19 and 22 (Alt Ribo, Figure 1E). Again, we observed a strong decrease in the formation of longer transcripts, which was supported by lane densitometry analysis (Supplementary Figures S1–S4). These results suggest that the presence of rNMPs in the DNA template reduces the processivity of POLRMT, and the transcription elongation factor TEFM cannot overcome this effect. Similar results were obtained with transcription factors from mice (Figure 1F).

### Embedded rNMPs impair transcription on double-stranded DNA

**Figure 2.**
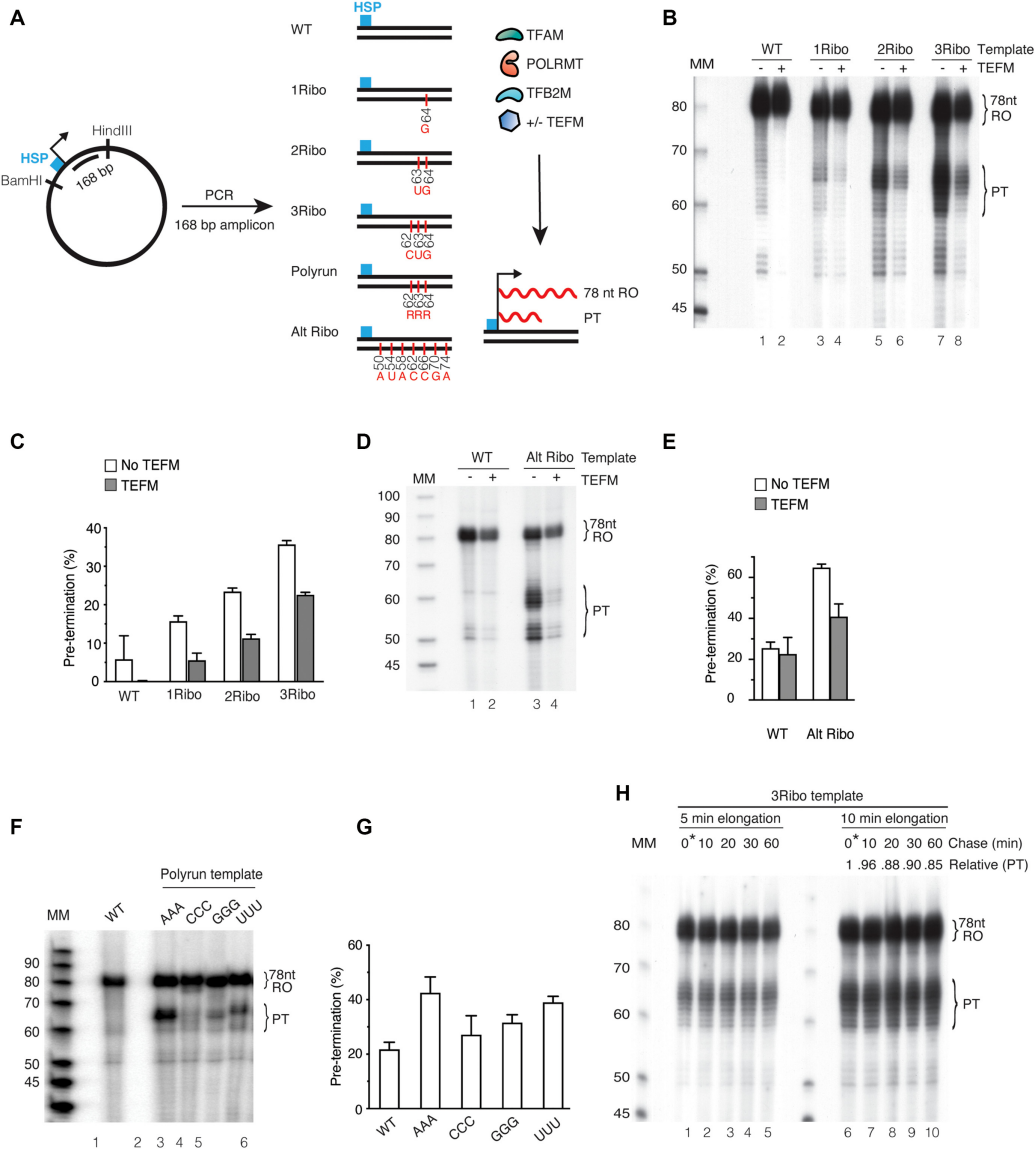
Pre-termination is increased in the presence of a ribonucleotide incorporated heavy strand promoter template. (**A**) Schematic representation of the template containing HSP. *In vitro* transcription reactions were performed in the presence of TFAM, POLRMT, TFB2M and with/without TEFM, allowing for the formation of a 78 nt RO product. A: adenine; G: guanine; C: cytosine; U: uracil. (**B**) *In vitro* transcription using ribonucleotide incorporated DNA templates 1Ribo-(**G**), 2Ribo-(**GU**) and 3Ribo-(**GUC**). TFAM, POLRMT and TFB2M were added in all lanes. The RO product and the pre-terminated transcript (PT) are indicated. Lane 1 is a control reaction performed with a WT template. (**C**) Densitometry quantification of RO product and PT from panel (B). Experiments were performed in triplicate and error bars depict the standard deviation. Pre-termination is represented as a percentage of total transcription (RO + PT). (**D**) *In vitro* transcription was performed using the DNA templates with ribonucleotides incorporated on every third position. The RO product and PT are indicated. (**E**) Densitometry quantification of RO product and PT from panel (D). Experiments were performed in triplicate and error bars show standard deviation. Pre-termination is represented as a percentage of total transcription (RO + PT). (**F**) *In vitro* transcription reactions using DNA templates with three identical, embedded rNMPs, 3Ribo-(**AAA**), -(**CCC**), -(**GGG**) and -(**UUU**) were performed in lanes 2–5, respectively. Lane 1 is a control reaction performed with a WT template. The RO product and PT are indicated. (**G**) Densitometry quantification of RO product and PT from panel (F). Experiments were performed in triplicate and error bars depict the standard deviation. Pre-termination is represented as a percentage of total transcription (RO + PT). (**H**) *In vitro* pulse-chase transcription reaction with 3Ribo-(**GUC**) template. Transcription elongation in lanes 1–5 was carried out for 5 min and in lanes 6–10 for 10 min, followed by the addition of excess of cold UTP simultaneously. Controls in lanes 1 and 6 were stopped immediately after adding an excess of cold UTP. Lanes 2 and 7 were stopped after 10 min, lanes 3 and 8 after 20 min, lanes 4 and 9 after 30 min, and lanes 5 and 10 after 60 min. The PT is quantified relative to the zero-time point. Reaction products were separated on a 10% denaturing polyacrylamide sequencing gel. Molecular weight is indicated (Affymetrix low MW 10–100 nt ladder).

POLRMT is non-processive on a single-stranded DNA template, which may aggravate the effect embedded ribonucleotides have on transcription when it is monitored on a ssDNA template (34). We thus investigated the effects of embedded ribonucleotides using double-stranded DNA fragments, with 1, 2 or 3 rNMPs at defined positions in the template strand (Figure 2A). The transcription reactions contained POLRMT, TFAM, TFB2M and a linearised, double-stranded DNA template containing HSP. In contrast to the primed single-stranded DNA template used in Figure 1, the double-stranded template could support multiple rounds of transcription.

We observed a 78 nt RO transcript and multiple shorter products with the WT template. In agreement with previous reports (15,18), addition of TEFM stimulated the formation of full-length products (Figure 2B, compare lanes 1 and 2) and removed a range of shorter, transcription products formed independently of embedded ribonucleotides. The 1Ribo template with a ribonucleotide G at position 64 in the template strand showed increased amounts of shorter transcripts with an apparent length of 63–67 nt (Figure 2B, lane 3), possibly corresponding to premature transcription termination (PT). This effect was accentuated with the 2Ribo (Figure 2B lane 5), 3Ribo (Figure 2B lane 7), and every third Ribo templates (Figure 2D, lanes 3 and 4). We also quantified the RO and PT products (Figure 2C, E and G) and calculated the percentage of pre-termination relative total transcription (PT/(RO + PT)). The levels of pretermination were relatively high, reaching about 40% on a template containing seven embedded ribonucleotides present on every third position in the template strand.

To further understand these results, we produced four different templates, each with a short poly-run of the same ribonucleotides (AAA, CCC, GGG, and UUU) (Figure 2F, lanes 2–5, quantified in Figure 2G). We observed premature termination with all templates, but the effect was strongest with the AAA template, whereas the CCC template had a milder effect.

### Embedded rNMPs cause pre-termination, not temporary pausing

To determine whether the shorter transcripts were indeed produced by premature transcription termination at embedded ribonucleotides or were due to pausing the transcription machinery, we performed a pulse-chase experiment (Figure 2H). Transcription was initiated in the presence of radiolabeled UTP. After 5 or 10 min, a 500-fold excess of cold UTP was added and the reactions proceeded until 60 min. If the shorter products result from POLRMT pausing, a progressive decrease in the ratio of PT to RO transcripts would be observed due to resumed elongation of stalled enzymes. However, the levels of PT transcripts remained unchanged for up to 60 min. Little variation in the relative PT from 0–60 min also supports the notion that the shorter products were formed by premature transcription termination, even if we cannot rule out permanent stalling.

### rNMPs embedded in the non-template strand do not affect transcription

**Figure 3.**
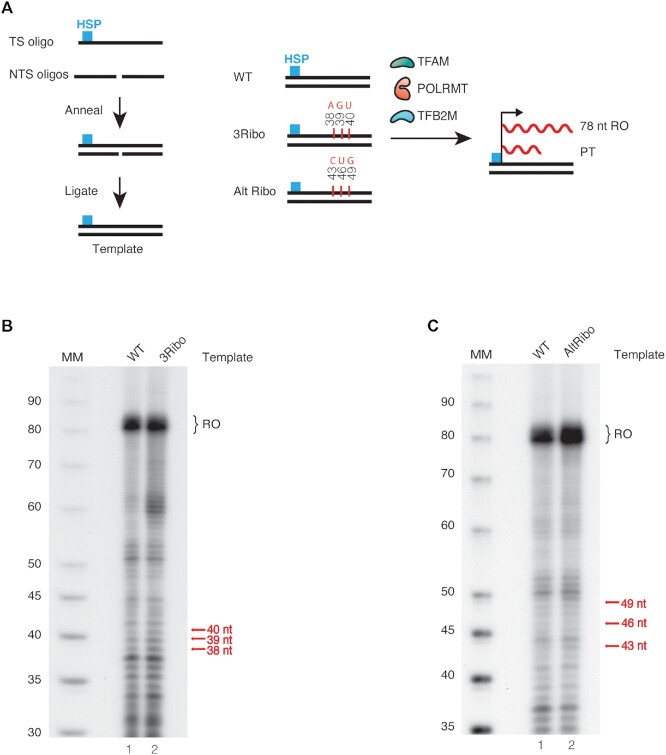
Ribonucleotides embedded in the non-template strand have limited effects on transcription elongation. A: adenine; G: guanine; C: cytosine; U: uracil. (**A**) Schematic representation of the template strand (160 nt long oligo) containing the promoter region and TFAM binding site. (**B**) *In vitro* transcription reaction performed using the ribonucleotide incorporated non-template strand, 3Ribo-(**AGU**). RO: run-off product. (**C**) *In vitro* transcription reaction performed using ribonucleotides-incorporated non-template strand, Alt Ribo-(**C**–**U**–**G**).

We investigated the effects of embedded ribonucleotides in the non-template strand (Figure 3A). These reactions were performed with two different templates, one carrying three consecutive ribonucleotides at positions 38–40, and a second in which rNMPs were incorporated at positions 43, 46 and 49. We observed a 78 nt RO product with both templates, but no significant increase in the level of pre-terminated transcripts at the expected positions (Figure 3B and C, quantified in Supplementary Figure S5A and B, respectively).

### Effects of embedded ribonucleotides on the T7 RNA polymerase

**Figure 4.**
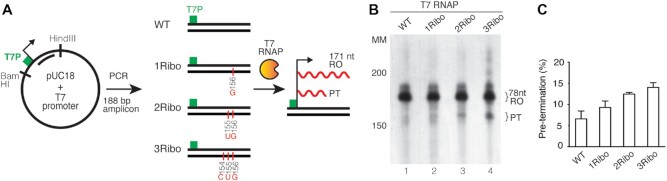
Pre-termination is not significantly increased in the presence of the T7 promoter and T7 RNA polymerase. (**A**) Schematic representation of the template used for transcription (similar to Figure 2A). 188 bp of the PCR product was amplified containing the T7 promoter region in the forward primer. A: adenine; G: guanine; C: cytosine; U: uracil. (**B**) *In vitro* transcription from the ribonucleotide incorporated DNA template in the presence of T7 RNA polymerase. Templates were used as described in Figure 2B. The RO and PT products are indicated. (**C**) Densitometry quantification of RO product and PT from panel (B). Experiments were performed in triplicate and error bars show standard deviation. The pre-termination is represented as a percentage of total transcription (RO + PT). The reaction products were run on a 4.5% denaturing polyacrylamide sequencing gel. Molecular weight is indicated (Affymetrix low MW 10–100 nt ladder).

T7 RNAP is structurally related to POLRMT. To investigate if the effects observed with embedded rNMPs were specific to POLRMT, we repeated our experiments with the phage enzyme. We introduced a T7 promoter upstream of HSP and performed *in vitro* transcription using templates containing 1, 2 or 3 ribonucleotides (Figure 4A). The expected RO transcript was 188 nt and rNMPs were located at positions 172–174 nt in the transcript formed (Figure 4B). Similar to POLRMT, we observed premature transcription termination, but the phage polymerase appeared less sensitive to embedded ribonucleotides (compare Figure 4B and C with Figure 2B and C), which is consistent with a previous report (35). Here, the level of premature transcription termination by T7 RNAP was <15% at a stretch of three consecutive ribonucleotides (Figure 4C).

### Substitutions in POLRMT that affect hydrogen bonding do not affect the pre-termination

**Figure 5.**
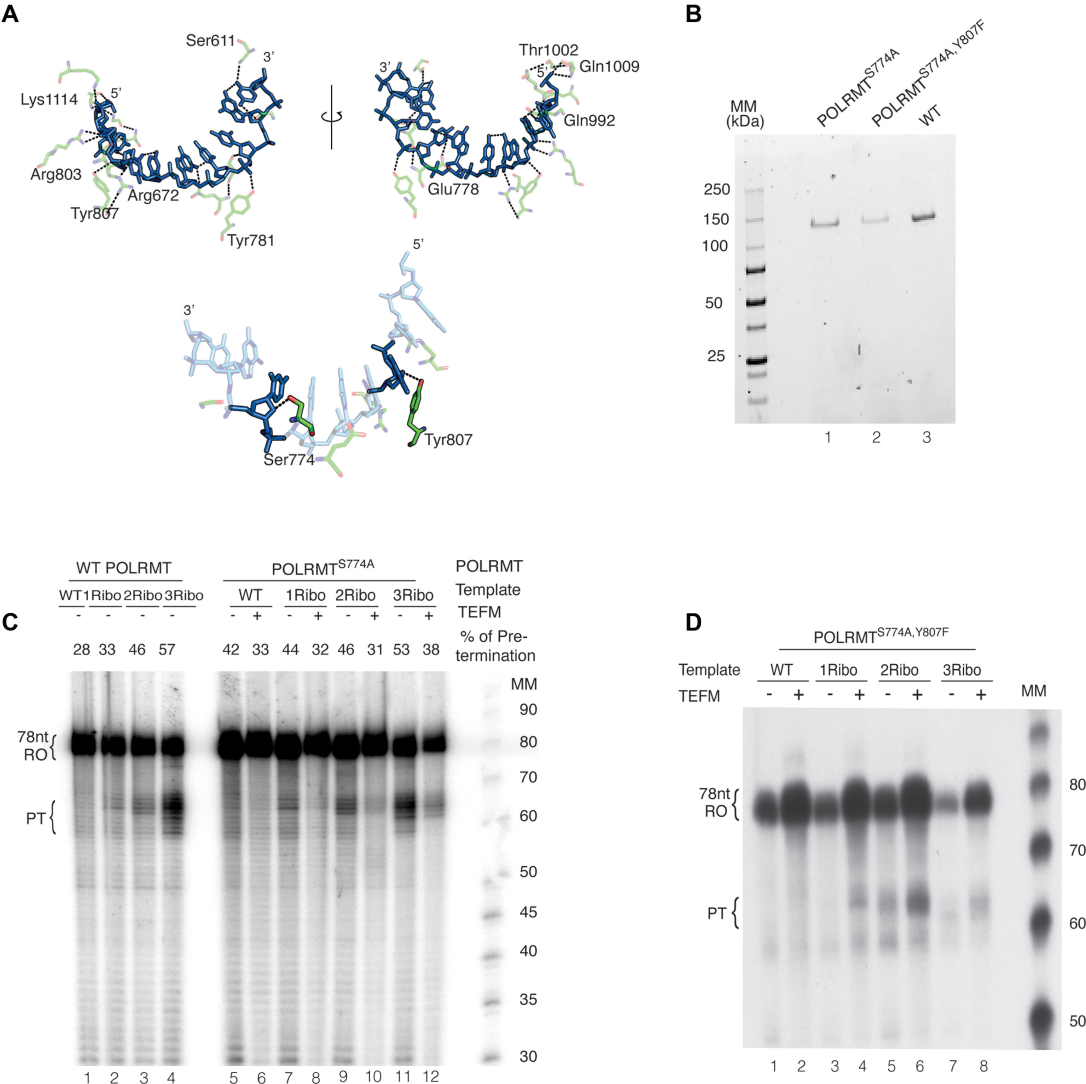
Ser774 and Tyr807 in POLRMT do not affect pre-termination at embedded rNMPs *in vitro*. (**A**) Serine and Tyrosine at 774th and 807th positions (Ser774 and Tyr807) in the thumb and palm region of C-terminal domain in POLRMT, respectively, were predicted to be responsible for transcription termination. (**B**) Proteins were expressed, purified, and shown in SDS-PAGE as recombinant mutant POLRMT single mutant S774A (POLRMT^S774A^), double-mutant S774A, Y807F (POLRMT^S774A,Y807F^), and WT in lanes 1, 2, and 3, respectively. Molecular weight (kDa) is indicated. (**C**) *In vitro* transcription from ribonucleotide incorporated DNA templates: 1Ribo-(**G**), 2Ribo-(**GU**), 3Ribo-(**GUC**). TEFM was added where indicated. Lanes 1–4 were performed with WT POLRMT and lanes 5–12 were performed with S774A mutant POLRMT. (**D**) *In vitro* transcription from ribonucleotide incorporated DNA templates, as in Figure 2B. TEFM was added where indicated. The reactions were run on 10% denaturing polyacrylamide sequencing gel. Molecular weight is indicated (Affymetrix low MW 10–100 nt ladder).

The structure of elongating POLRMT suggests that hydrogen-bonding (H-bonding) occurs between the hydroxyl groups of amino acids Ser774 and Tyr807 and the 2′-carbon proton of deoxyribose (Figure 5A) (36). We speculated that loss of this interaction could explain the premature transcription termination effect, since the ribose sugar possesses a hydroxyl group on its 2′-carbon. Single and double mutants of POLRMT were created where Ser774 and Tyr807 were replaced with alanine and phenylalanine, respectively, thereby eliminating the H-bonding hydroxyl groups (Figure 5B). We could efficiently express and purify POLRMT^S774A^ and could obtain low levels of POLRMT^S774A, Y807F^, but we were unable to produce soluble POLRMT^Y807F^. An *in vitro* transcription reaction showed no differences in PT transcript formation with the POLRMT^S774A^ (Figure 5C, Supplementary Figure S6). The mutant version with two mutations, POLRMT^S774A,Y807F^ was less active in transcription, but this did not lead to apparent changes in relative PT levels (Figure 5D).

## DISCUSSION

The mitochondrial DNA polymerase POLγ can efficiently discriminate between rNTPs and dNTPs during DNA replication. *In vitro* analysis indicates that POLγ introduces one ribonucleotide for every 2.0 ± 0.6 × 10^3^ dNTPs. However, due to the absence of active ribonucleotide excision repair in mitochondria, incorporated ribonucleotides will not be repaired, but remain in replicated mtDNA (21–23). In agreement with this notion, mtDNA is rich in rNMPs and sensitive to alkaline hydrolysis. In cell lines, there are up to 54 rNMPs per 16 kb mammalian mtDNA molecule. DNA fragmentation analysis has demonstrated that the levels of embedded rNMPs are even higher *in vivo* in postmitotic tissues, but the exact frequency is not known (21,23). POLγ can efficiently bypass a single embedded rNMP in template DNA, but this is diminished when a stretch of multiple ribonucleotides is present in template DNA. It is possible that such stretches of ribonucleotides may contribute to disease phenotypes (22,23).

In this work, we investigated the effects of embedded ribonucleotides on transcription. Our data demonstrate that POLRMT has problems bypassing a single embedded rNMP in template DNA, and the efficiency is further diminished when a stretch of two or three ribonucleotides are present. Pre-termination is also observed when embedded ribonucleotides are present at every third position. TEFM slightly lowers this effect, but substantial levels of pretermination are still observed. By using pulse chase labelling we can conclude that the observed effect is due to true transcription termination and not by POLRMT pausing.

Our data suggest that ribonucleotides embedded into template DNA have a deleterious effect on mitochondrial transcription *in vitro*. We find it very likely that the same effects will be observed *in vivo*, even if this remains to be demonstrated. Other factors, such as nucleoid formation and negative supercoiling, could for instance affect the number of transcription termination events. The effects described here could potentially contribute to disease phenotypes associated with disturbed nucleotide pools, since such conditions are associated with higher levels of embedded ribonucleotides (21). It is conceivable that higher levels of rNMPs in mtDNA may have a double hit effect: first, impairing DNA replication and thereby lowering mtDNA levels, and second, reducing full length transcription of mtDNA. It is also worth noting that we observe a mild level of transcription termination at even a single embedded rNMP. Multiple single rNMPs present on a DNA template strand could lead to a substantial effect on the fraction of transcription initiation events that form full length transcription products.

Initially, we speculated that the observed effects could be due to direct interactions between POLRMT and embedded ribonucleotides. In support of this notion, we observed that transcription often terminated 1–3 nucleotides downstream of the embedded ribonucleotide. Structural analysis suggested that amino acids Ser774 and Tyr807 could form H-bonds with the hydrogen of the 2′-carbon of deoxyribose in the template strand at positions +7 and +3, respectively, thus potentially sensing the presence of rNMPs in the template strand. However, non-H-bonding mutations of these amino acids did not affect the transcription termination at embedded ribonucleotides. Interestingly, recent structural analysis of yeast mitochondrial RNA polymerase demonstrates that Tyr831, which is analogous to human Tyr807, interacts with the base and not with the sugar, which could explain the lack of an effect observed here (37). Presently, our working model is that the transcription termination observed here is due to structural changes in the template. Others have demonstrated that single ribonucleotide incorporation into DNA perturbs the structure of both strands in a double helix on the 3′ side of the lesion (38). However, structural modeling of elongating RNA polymerase II did indicate that a single, embedded ribose only has minor effects on template strand geometry at the active site and transcription elongation (39). Going forward, structural characterization of elongating POLRMT in complex with templates containing one or more embedded ribose residues may help to address this question.

## DATA AVAILABILITY

Raw data are available from the corresponding author upon reasonable request.

## Supplementary Material

gkab1251_Supplemental_File

## Notes

Present address: Maria Falkenberg, Department of Clinical Pathology, Sahlgrenska University Hospital, Gothenburg, Sweden.

Present address: Claes M. Gustafsson, Department of Clinical Chemistry, Sahlgrenska University Hospital, Gothenburg, Sweden.
