# Correction to ‘ribonucleotides embedded in template DNA impair mitochondrial RNA polymerase progression’

**DOI:** 10.1093/nar/gkad1169

**Published:** 2023-12-12

*Nucleic Acids Research*, Volume 50, Issue 2, 25 January 2022, Pages 989–999, https://doi.org/10.1093/nar/gkab1251

The authors wish to correct an error in their article where they erroneously misassigned the position on the template DNA involved in H-bond formation with the template strand.

The following sentences in the result section:

The structure of elongating POLRMT suggests that hydrogen-bonding (H-bonding) occurs between the hydroxyl groups of amino acids Ser774 and Tyr807 and the **2′-carbon proton of deoxyribose** (Figure5A) (36). We speculated that loss of this interaction could explain the premature transcription termination effect, **since the ribose sugar possesses a hydroxyl group on its 2′-carbon**.

Have been corrected to:

The structure of elongating POLRMT suggests that hydrogen-bonding (H-bonding) occurs between the hydroxyl groups of amino acids Ser774 and Tyr807 and the **the oxygen in the ribose/deoxyribose ring at position + 7 and + 3 in the template strand** (Figure 5A) (36). We speculated that loss of this interaction could explain the premature transcription termination effect.

The following sentence in the discussion:

Structural analysis suggested that amino acids Ser774 and Tyr807 could form H-bonds with **the hydrogen of the 2′-carbon of deoxyribose** in the template strand at positions + 7 and + 3, respectively, **thus potentially sensing the presence of rNMPs in the template strand**. However, non-H-bonding mutations of these amino acids did not affect the transcription termination at embedded ribonucleotides.

Has been corrected to:

Structural analysis suggested that amino acids Ser774 and Tyr807 could form H-bonds with **the oxygen in the ribose/deoxyribose ring** in the template strand at positions + 7 and + 3, respectively. However, non-H-bonding mutations of these amino acids did not affect the transcription termination at embedded ribonucleotides.

The structure is unchanged, and Figure 5 remains valid. This correction does not affect the results, discussion and conclusions presented in the article. These details have been corrected only in this correction notice to preserve the published version of record.

