# Supplementary material for: Ribonucleotides embedded in template DNA impair mitochondrial RNA polymerase progression
Source: Nucleic Acids Res. 2022 Jan 8;50(2):989–99. doi: 10.1093/nar/gkab1251 (PMC8789056; doi:10.1093/nar/gkab1251)
Supplement: gkab1251_Supplemental_File [file gkab1251_supplemental_file.pdf]

SUPPLEMENTARY INFORMATION FOR

Ribonucleotides embedded in template DNA impair mitochondrial RNA polymerase progression

Meenakshi Singh[1], Viktor Posse[2], Bradley Peter[1], Maria Falkenberg[1], Claes M. Gustafsson[1]*

[1]Institute of Biomedicine, University of Gothenburg, Gothenburg, SE-405 30, Sweden

[2]Department of Medical Biochemistry and Cell Biology, University of Gothenburg, Gothenburg, SE-405 30, Sweden

* To whom correspondence should be addressed.

Email: claes.gustafsson@medkem.gu.se

Tel: +46-70-8589521

Supplementary Figures

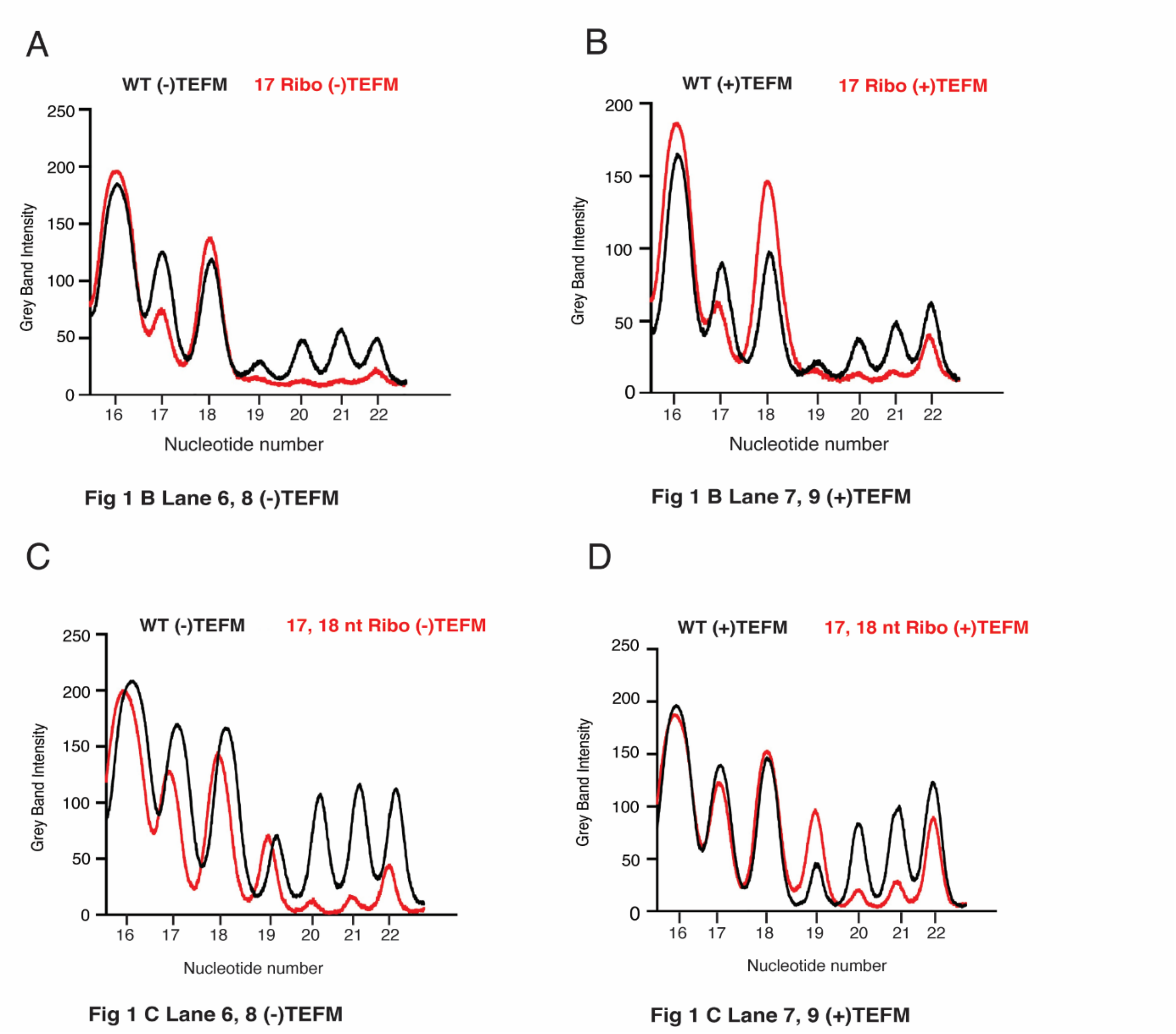

Figure S1. Lane densitometric quantification of *in vitro* transcription reactions performed in Figure 1B and 1C. **(A)** Lane densitometry of Figure 1B, lane 6 and 8 (without TEFM). **(B)** Lane densitometry of Figure 1B, lane 7 and 9 (with TEFM). **(C)** Lane densitometry of Figure 1C, lane 6 and 8 (without TEFM). **(D)** Lane densitometry of Figure 1C, lane 7 and 9 (with TEFM).

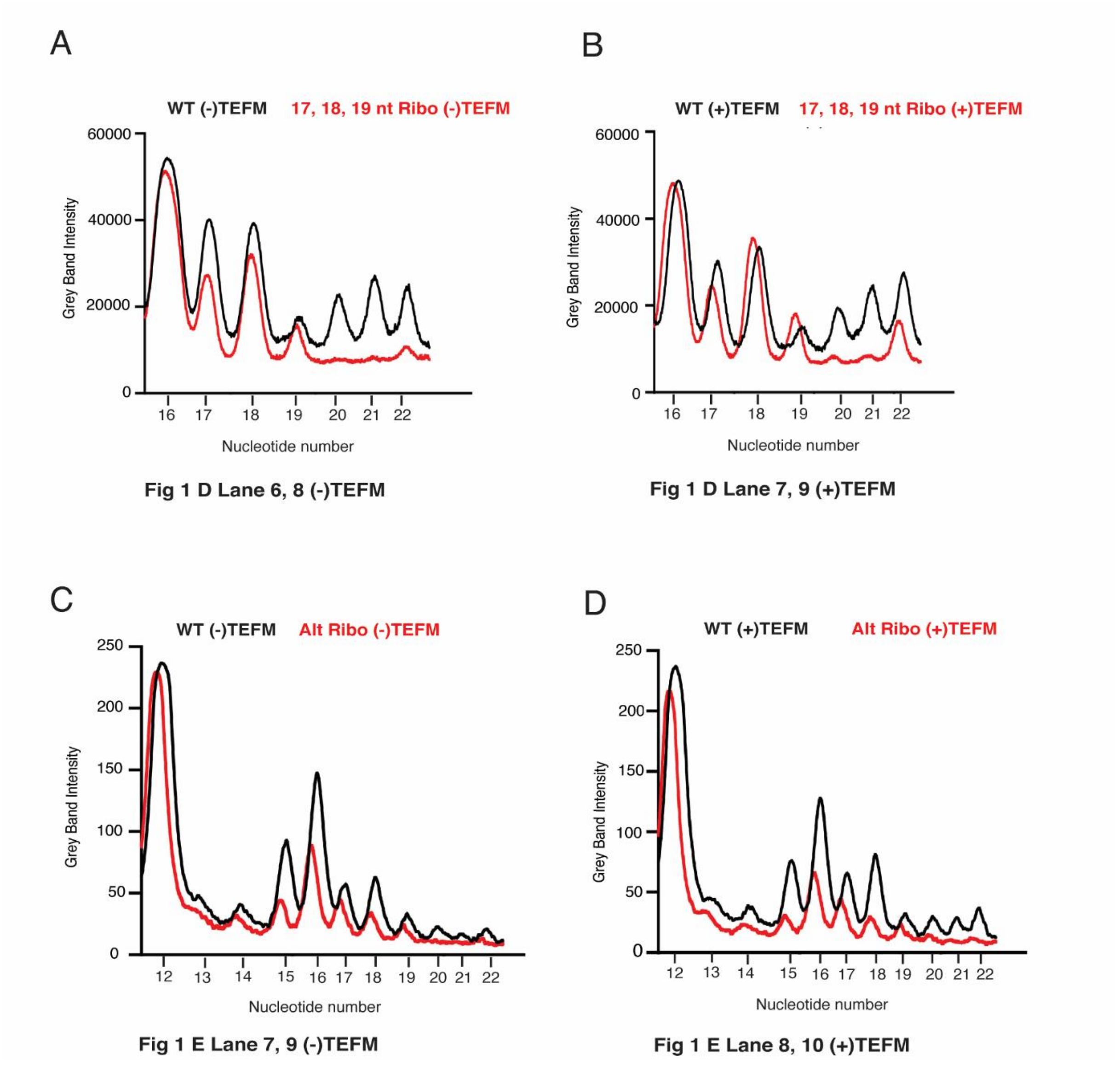

Figure S2. Lane densitometric quantification of *in vitro* transcription reactions performed in Figure 1D and 1E. **(A)** Lane densitometry of Figure 1D, lane 6 and 8 (without TEFM). **(B)** Lane densitometry of Figure 1D, lane 7 and 9 (with TEFM). **(C)** Lane densitometry of Figure 1E, lane 7 and 9 (without TEFM). **(D)** Lane densitometry of Figure 1C, lane 8 and 10 (with TEFM).

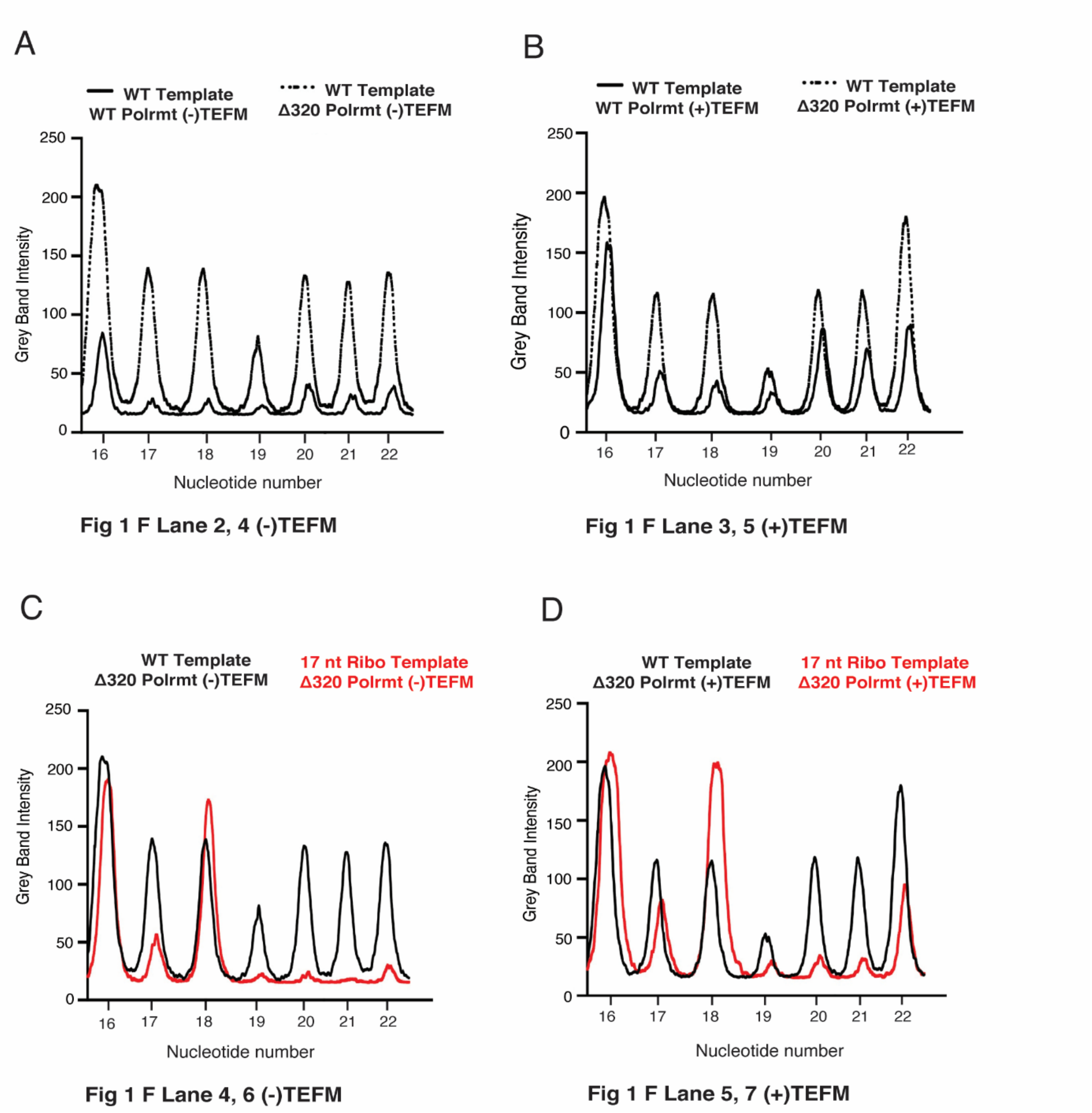

Figure S3. Lane densitometric quantification of *in vitro* transcription reactions performed in Figure 1F. **(A)** Lane densitometry of Figure 1F, lane 2 and 4 (without TEFM). **(B)** Lane densitometry of Figure 1F, lane 3 and 5 (with TEFM). **(C)** Lane densitometry of Figure 1F, lane 4 and 6 (without TEFM). **(D)** Lane densitometry of Figure 1F, lane 5 and 7 (with TEFM).

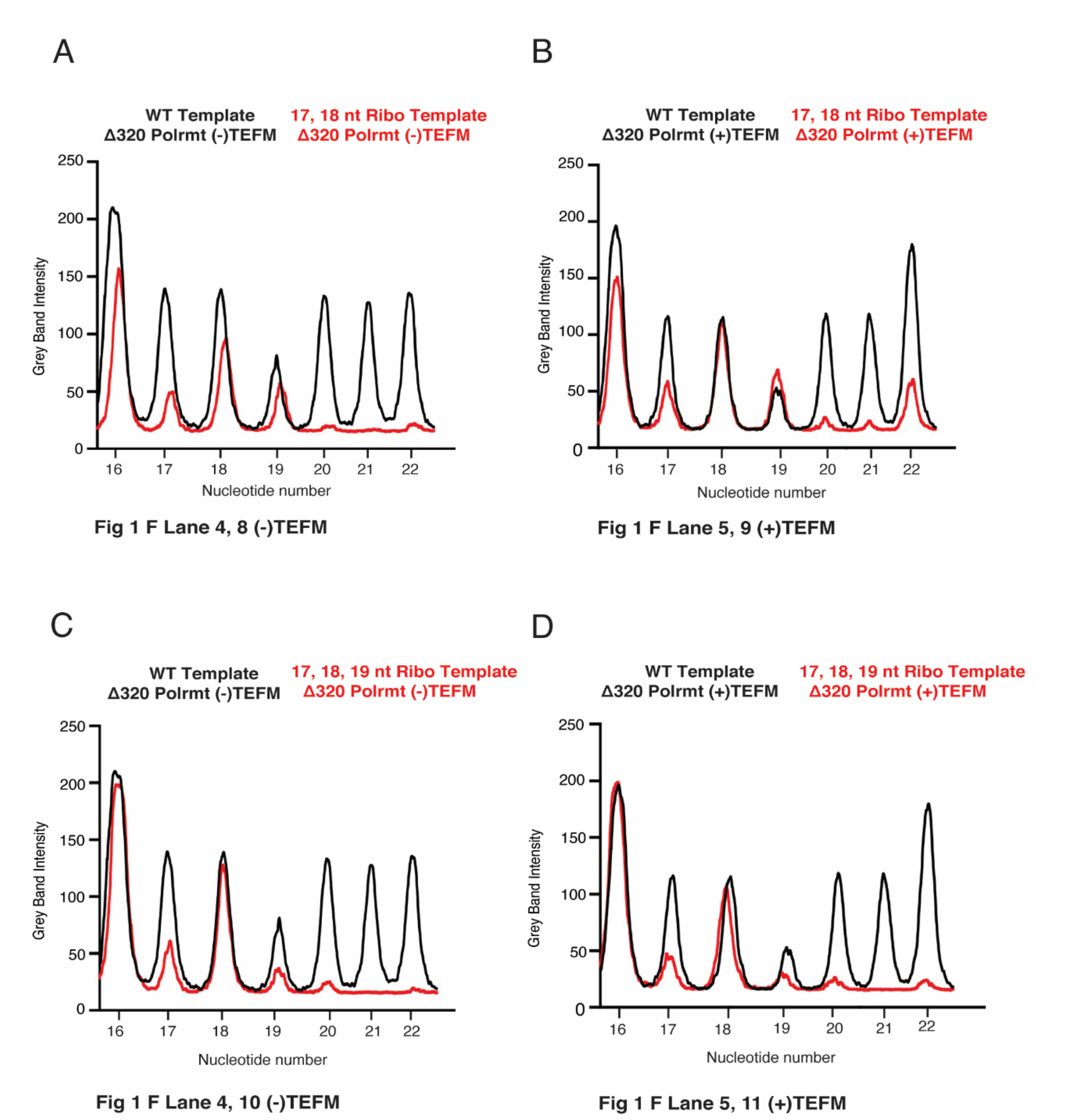

Figure S4. Lane densitometric quantification of *in vitro* transcription reactions performed in Figure 1F. **(A)** Lane densitometry of Figure 1F, lane 4 and 8 (without TEFM). **(B)** Lane densitometry of Figure 1F, lane 5 and 9 (with TEFM). (C) Lane densitometry of Figure 1F, lane 4 and 10 (without TEFM). (D) Lane densitometry of Figure 1F, lane 5 and 11 (with TEFM).

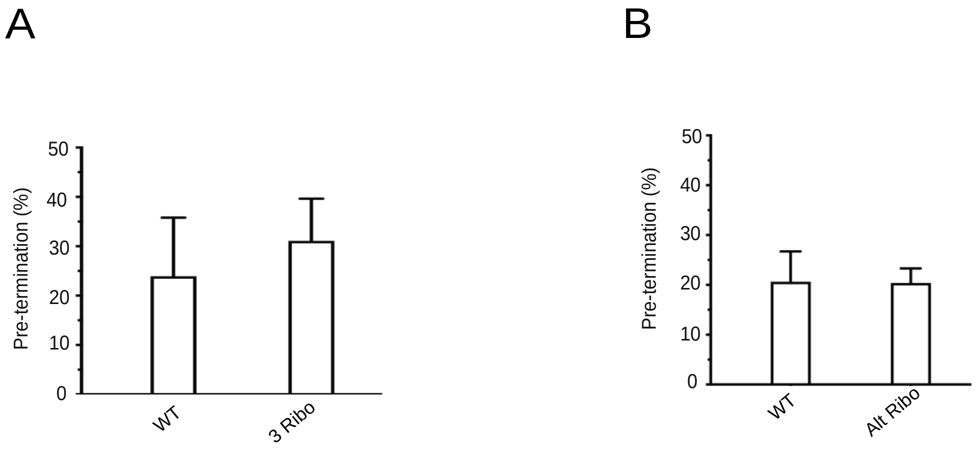

Figure S5. Densitometry quantification of RO product and PT performed in **(A)** Figure 3B and **(B)** 3C. Experiments were performed in triplicate and error bars depict the standard deviation. Pre-termination is represented as a percentage of total transcription (RO + PT).

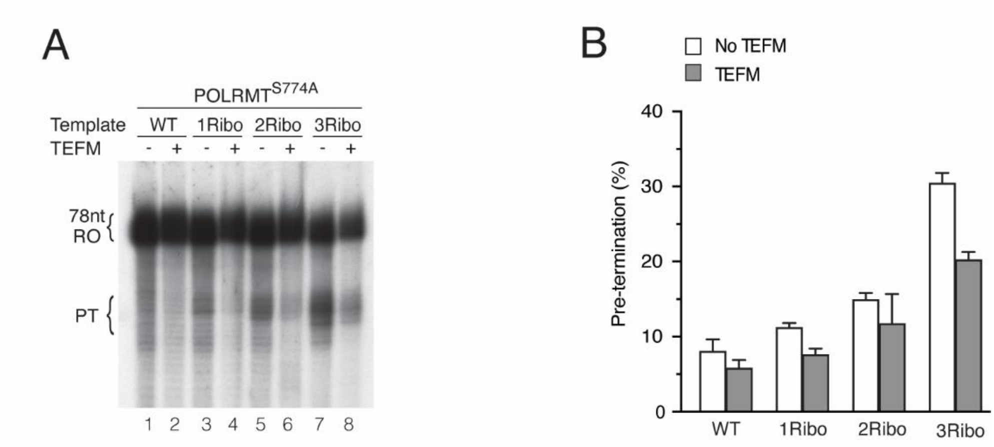

Figure S6. (A) *In vitro* transcription from ribonucleotide-incorporated DNA templates: 1Ribo-(**G**), 2Ribo-(**GU**), 3Ribo-(**GUC**). TEFM was added where indicated. Reactions were performed with S774A mutant POLRMT. The reaction product was run on a 10% denaturing polyacrylamide sequencing gel. **(B)** Densitometry quantification of RO product and PT from (A). Experiments were performed in triplicate and error bars show standard deviation. The pre-termination is represented as a percentage of total transcription (RO + PT).
